# Christ's Hospital and St. Bartholomew's

**Published:** 1900-11-10

**Authors:** 


### CHRIST'S HOSPITAL AND ST. BARTHOLOMEW'S.

The story of the haggling which has of late been going on between the authorities of Christ's Hospital on the one hand and those of St. Bartholomew's Hospital on the other, is not an altogether agreeable one, and must raise many questions in the minds of those who have to deal with public bodies. If it is to be maintained that those who act as trustees for public institutions are to regard themselves as always bound to demand their pound of flesh, while, owing to the constant changes of their personnel they are excused from being tied down by arrangements which in ordinary business matters would be held binding between man and man, civic life in London, centreing as it does on the proceedings of ancient corporations and public bodies, will become intolerable.

The position seems to be this. For years past it has been known that sooner or later Christ's Hospital must go out into the country, and for years past St. Bartholomew's has seen that to meet the growing demands upon it an enlargement of its site would be of the greatest service, if not indeed absolutely necessary to it, in carrying on its charitable work. Hence the subject of the acquisition of the site of Christ's Hospital by St. Bartholomew's has again and again been discussed between the managers of the two charities, with the result that an engagement was entered into in the year 1893, as we understand at the voluntary suggestion of the Council of Christ's Hospital, to the effect that when the school went into the country St. Bartholomew's should have such portion of the site as it should require, and that the price to be paid should be settled by some form of arbitration which should be fair to both parties.

This was quite sufficient for the authorities of St. Bartholomew's. The matter was not immediately urgent; all they wanted was to be assured that when the time came they should have the land, and that the price should be fixed by a satisfactory arbitration; so they waited until, in the fulness of time, the site was at liberty, relying on an engagement which they still consider binding on the Council of Christ's Hospital as men of honour. With flux of time, however, changes have occurred, and the present Council apparently decline to be so bound. Posing as trustees, whose duty they take it is to obtain the uttermost farthing for everything they sell, they are reported to be in negotiation with a speculative syndicate for the sale of the whole property, the only protection given to St. Bartholomew's being the insertion, in the agreement for the sale, of a provision that the purchaser shall resell to St. Bartholomew's all that portion of the site which is to the north of a certain line, and is contiguous to the hospital property. Truly, this includes the bit of land in regard to which St. Bartholomew's has been most anxious to negotiate—the minimum of its requirements; but the minimum does not express all that the hospital really wants, nor all that it hoped to obtain under the old but now repudiated engagement; while in regard to anything over this line the agreement leaves the hospital at the mercy of the speculative syndicate, a prospect which is not altogether satisfactory. Again, this clause provides that St. Bartholomew's is to pay for this "back" land a price which is to be *pro ratâ* of the whole—in other words is to pay for "back" land of irregular shape and but little frontage at the same price as is paid for some of the most valuable frontage land in the City of London. No wonder that the authorities of St. Bartholomew's who had relied upon the arrangement entered into seven years ago for them to have what they wanted at a price to be fixed by arbitration, object to being handed over to the tender mercies of a speculative syndicate. However there is a Parliament to which appeal may be made, and probably will be made, in order that St. Bartholomew's may obtain power for the compulsory purchase of the land which it requires. But it is a sorry spectacle, and indeed to many it must seem almost inconceivable that in the City of London a public body like the Council of Christ's Hospital, in the teeth of what many business men regard as a solemn promise, should refuse to sell the land required for hospital purposes by one of our greatest charities. We understand that the authorities of St. Bartholomew's Hospital have taken the preliminary steps necessary to acquire the land compulsorily, and seeing that they are quite prepared to pay its full value—whatever price competent authorities may put upon it—we certainly shall be surprised if Parliament does not regard the claims of one of London's greatest charities as of more importance than this strange contract between "Christ's" Hospital and a speculative syndicate.

